# Lymphoma of the Base of the Tongue: An Incidental Finding on the Preoperative Workup for a Spine Surgery

**DOI:** 10.4021/jocmr2009.07.1251

**Published:** 2009-08-20

**Authors:** Mohammad Sami Walid

**Affiliations:** Medical Center of Central Georgia, 840 Pine Street, Suite 880, Macon, GA 31201, USA. Email: mswalid@yahoo.com

## Abstract

Lymphoma of the tongue is very rare and accounts for 1% of all malignant tumors of the oral cavity. We present a patient who presented with weakness and difficulties with ambulation who was a known cervical spine spondylosis and stenosis patient. During the preoperative workup for cervical spine surgery a tumor at the base of the tongue was discovered which was confirmed by pathology to be a lymphoma.

**Keywords:**

Lymphoma; Tongue; Spine surgery; Preoperative workup

## Case Report

**Figure 1:**
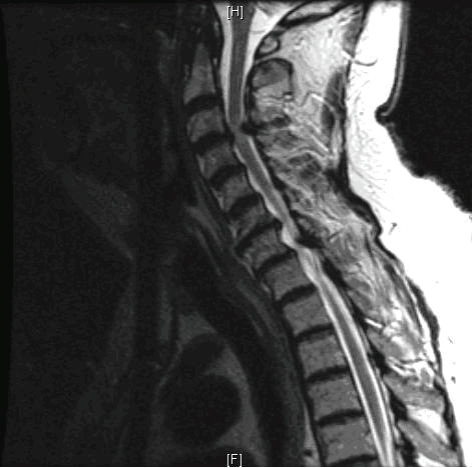
MRI T2 of the cervical spine showing multilevel spondylosis and moderate cord impingement at C3-C4.

Lymphoma of the tongue is very rare and accounts for 1% of all malignant tumors of the oral cavity [1-5]. We present a 73 year-old Caucasian man with history of hypertension, chronic obstructive pulmonary disease, and hyperlipidemia who presented in 2008 because of weakness and was ambulating using a walker. In 2004, he was diagnosed with multilevel cervical spondylosis, moderate spinal stenosis at C3-C4 with bilateral foraminal stenosis and central disk protrusion at C5-C6. He was also found to have a moderate-to-severe stenosis at L3-L5. He was a lifelong smoker with nearly a 100 pack-year history. On admission he had low sodium (128 mEq/L). A postadmission MRI shows a moderately advanced multilevel spondylosis most pronounced at C3-C4 with prominent disc bulge causing moderate cord impingement (Fig. 1). The myelogram shows an apparent block in the central spinal canal at the C3-C4 level with grade I anterolisthesis at the same level and degenerative changes throughout the mid and lower cervical spine.

**Figure 2:**
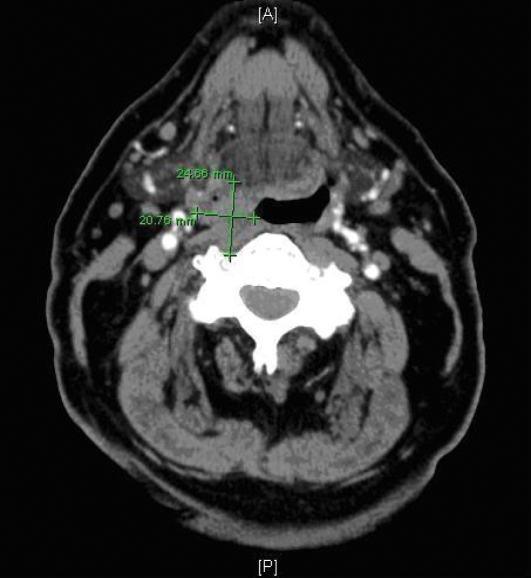
Neck CT with contrast showing a soft density mass in the right hypopharynx lateral wall extending around the lateral pharyngeal wall into the prevertebral soft tissues on the right.

During the preoperative medical exam, the patient states he has been also having sore throat, night sweats, and shortness of breath. For the sore throat, an Ear-Nose-Throat consultation is requested. Laryngoscopy shows a smooth mucosa-covered mass in the right half of the base of the tongue. Pathological study of the biopsy reveals grade II/III follicular lymphoma, positive for B cell clonality. Neck CT is ordered which shows a soft tissue mass in the right hypopharynx extending from the base of the tongue into the posterior prevertebral musculature (Fig. 2). Scattered lymph nodes along the internal jugular chain on the right as well as nodes around the submandibular gland are noticed on CT, however, none of these exceeds a centimeter in diameter and there are almost as many subcentimeter nodes in the same location on the left side. The patient undergoes anterior cervical decompression and fusion at C3-4 and is discharged to follow up with otolaryngology.

In this patient, the weakness was caused by the cervical canal stenosis and worsened by hyponatremia as a sign of a paraneoplastic syndrome caused by the lymphoma. This case illustrates the importance of performing laryngoscopy in elderly patients during the preoperative workup for any major surgery if they complain of sore throat and shortness of breath with a lifelong history of smoking [1]. Unfortunately, we read our patients obituary in the local newspaper eight months later.
